# A French national breast and thyroid cancer screening programme for survivors of childhood, adolescent and young adult (CAYA) cancers - DeNaCaPST programme

**DOI:** 10.1186/s12885-017-3318-1

**Published:** 2017-05-12

**Authors:** Charlotte Demoor-Goldschmidt, Delphine Drui, Isabelle Doutriaux, Gérard Michel, Pascal Auquier, Agnès Dumas, Claire Berger, Valérie Bernier, Sandrine Bohrer, Pierre-Yves Bondiau, Bruno Filhon, Brice Fresneau, Claire Freycon, Dinu Stefan, Sylvie Helfre, Angela Jackson, Christine Kerr, Anne Laprie, Julie Leseur, Marc-André Mahé, Caroline Oudot, Claire Pluchard, Stéphanie Proust, Hélène Sudour-Bonnange, Céline Vigneron, Nathalie Lassau, Martin Schlumberger, Cécile Faure Conter, Florent de Vathaire

**Affiliations:** 1 Centre for Research in Epidemiology and Population Health (CESP), Cancer and Radiation team, INSERM U1018, Université Paris-Sud, UVSQ, Université Paris-Saclay, 94807 Villejuif, France; 2 Department of endocrinology, CHU de Nantes, 44000 Nantes, France; 3 Department of radiology, Institut de Cancérologie de l’Ouest – René Gauducheau, 44800 Saint Herblain, France; 4 Service d’hématologie et oncologie pédiatrique, Hôpital d’enfants La Timone, Marseille, France; 5 Unité de recherche EA 3279, Université Aix-Marseille, Marseille, France; 6 Service de santé publique, assistance publique – hôpitaux de Marseille et université Aix-Marseille, Marseille, France; 7 Department of Clinical Research, Gustave Roussy, 94805 Villejuif, France; 8 Claire Berger, hemato-oncology pediatric department, chu nord st Etienne, cedex, 42055 St Etienne, France; 9 Department of Radiation Oncology, Institut de Cancérologie de Lorraine, Nancy, France; 10 Oncology and Hematology Unit, CHU de Saint Denis de La Réunion, Saint Denis, France; 11 Department of Radiotherapy, Centre Antoine Lacassagne, Nice, France; 12 Department of Pediatric Hematology and Oncology, Rouen University Hospital, Rouen, France; 13 Pediatric oncology department, Gustave Roussy, Université Paris-Saclay, F-94805 Villejuif, France; 14 Service d’hématologie et d’oncologie pédiatrique du CHU de Grenoble, Grenoble, France; 15 Department of Radiation Oncology, Centre François Baclesse, Caen, France; 16 Department of Radiation Oncology, institut Curie, Paris, France; 17 Department of Radiation Oncology, institut du cancer de Montpellier, Montpellier, France; 18 Department of Radiation Oncology, IUCT Oncopole, Toulouse, France; 19 Department of Radiation Oncology, centre Eugène-Marquis, Rennes, France; 20 Department of Radiation Oncology, ICO, Nantes, France; 21 Pediatric Oncology Department, Hôpital de la Mère et de l’Enfant, 87042 Limoges, France; 22 Pediatric Oncology Department, chu Reims, hôpital américain, Reims, France; 23 Pediatric Oncology Department, chu Angers, Angers, France; 24 Pediatric Oncology Unit, Anti Cancer Center Oscar Lambret, cedex, 59020 Lille, France; 25 Department of Radiation Oncology, Centre de lutte contre le Cancer Paul Strauss, Strasbourg, France; 26 Imaging Department, Gustave Roussy Cancer Campus Grand Paris, IR4M UMR8081, Université Paris Sud, Villejuif, France; 27 Department of Nuclear Medicine and Endocrine Oncology, Gustave Roussy and Université Paris Saclay, 94805 Villejuif, France; 28 Pediatric Oncology Department, Centre Léon-Bérard, Lyon, France

**Keywords:** Childhood cancer, Survivor, Childhood cancer survivor, Breast cancer screening, Thyroid cancer screening, Breast, Thyroid, Second cancer, Secondary cancer

## Abstract

**Background:**

Survival of childhood, adolescent and young adult (CAYA) cancers has increased with progress in the management of the treatments and has reached more than 80% at 5 years. Nevertheless, these survivors are at great risk of second cancers and non-malignant co-morbidities in later life. DeNaCaPST is a non-interventional study whose aim is to organize a national screening for thyroid cancer and breast cancer in survivors of CAYA cancers. It will study the compliance with international recommendations, with the aim, regarding a breast screening programme, of offering for every woman living in France, at equal risk, an equal screening.

**Method:**

DeNaCaPST trial is coordinated by the INSERM 1018 unit in cooperation with the LEA (French Childhood Cancer Survivor Study for Leukaemia) study’s coordinators, the long term follow up committee and the paediatric radiation committee of the SFCE (French Society of Childhood Cancers).

A total of 35 centres spread across metropolitan France and la Reunion will participate. FCCSS (French Childhood Cancer Survivor Study), LEA and central registry will be interrogated to identify eligible patients. To participate, centers agreed to perform a complete “long-term follow-up consultations” according to good clinical practice and the guidelines of the SFCE (French Society of Children Cancers).

**Discussion:**

As survival has greatly improved in childhood cancers, detection of therapy-related malignancies has become a priority even if new radiation techniques will lead to better protection for organs at risk. International guidelines have been put in place because of the evidence for increased lifetime risk of breast and thyroid cancer. DeNaCaPST is based on these international recommendations but it is important to recognize that they are based on expert consensus opinion and are supported by neither nonrandomized observational studies nor prospective randomized trials in this specific population. Over-diagnosis is a phenomenon inherent in any screening program and therefore such programs must be evaluated.

**Electronic supplementary material:**

The online version of this article (doi:10.1186/s12885-017-3318-1) contains supplementary material, which is available to authorized users.

## Background

Survival of childhood, adolescent and young adult (CAYA) cancers has increased with progress in the management of the treatments and has reached more than 80% at 5 years [1, 2]. Nevertheless, these survivors are at great risk of second cancers and non-malignant co-morbidities in later life [3, 4]. There is paucity of information in the literature regarding tertiary prevention of secondary cancers.

Radiation therapy during childhood or young adulthood is an established risk factor for second breast cancer (SBC) [5]. Cohort studies have shown the cumulative risk of breast cancer to be approximately 10–33%, depending on the dose received by the breast, compared with a lifetime risk in the general population of 11–12%. This increased risk is mostly linearly dependent on the radiation dose received to the breast during radiation therapy, the shape of the dose response being modified by age at exposure and by early menopause [6–16]. The risk is higher with high dose of radiotherapy delivered at a young age*,* but is still significant after moderate (3–10 Gy) radiation dose [6, 10, 15]. The definition of a threshold dose is complicated because it depends on other parameters such as the size of the field, the age at treatment and hormonal status. The cumulative risk for a breast cancer after a mediastinal irradiation above 20 Gy is similar to that for BRCA2-mutated women: around 35% at 40 years [17]. Some studies suggest that these cancers are diagnosed at a median age of 35 years, and that they are more often bilateral hormone receptor-negative and high-grade compared with sporadic primary breast cancers [18, 19].

In CAYA cancer survivors, breast cancer screening is the subject of numerous articles, even in this specific population. Recently, Hodgson et al. presented the results of a mathematical model used to evaluate the marginal benefit on SBC mortality of early-initiated breast screening starting at age 25 years compared with screening initiated at age 40 years, which would be even later in France without any organized program (national breast screening starts at the age of 50) [20]. Their findings indicate that early MRI-based screening (starting at the age of 25 years) should reduce SBC mortality at the age of 75 from 16.65% with no early screening to 15.38% in the case of same-day annual mammography and MRI (16.28% with annual mammography, 15.40% annual MRI), leading to prevention of one SBC death for every approximately 80 patients screened. Many institutions, COG (Children’s Oncology Group), UK CCSG (Children’s Cancer and Leukaemia Group), NCI (National Cancer Institute), ACS (American Cancer Society) and HAS (French High Authority of Health), recommend breast screening for women at high and early risk; nevertheless, in the absence of any national program, these recommendations are not followed [21–23]. A harmonization has recently been published and recommends screening for women who have been exposed to chest irradiation above 20 Gy [24]. This screening should start at a minimum age of 25 years and a minimum delay after radiation of 8 years. Apart from the dose, the authors suggest taking into account other factors such as the size of the field, the family history of breast and ovarian cancer, in order to accurately evaluate the risk. The surveillance modalities are still under discussion. Most authors agree on an annual examination based on MRI, but the place of mammography, which delivers additional low doses of radiation, is still debated [18, 25–27].

The second most frequent cancer described in this population is thyroid cancer. Irradiation in childhood or young adulthood increases the risk of nodules and papillary cancers [28]. The time to onset is usually between 10 and 20 years after radiation exposure, but it has been described earlier or later. The best screening method for irradiated thyroid is still debated. The Scottish Intercollegiate Guidelines Network 2013, based on articles published before 2000, does not recommend systematic screening because of lack of clinical relevance. Nevertheless, an Italian prospective study of 129 subjects with a past history of thyroid irradiation who were monitored by ultrasound every three years found that three of the five patients with confirmed papillary carcinoma had non-palpable nodules at time of diagnosis, and advocated a systematic US-type surveillance [29]. The size of the nodule does not appear to be correlated with the risk of malignancy, but this risk does increase with an increase in the number of nodules [28]. In a retrospective study in Chicago on 1059 cancer survivors who have had a thyroidectomy because of a palpable nodule or radiological abnormality, the risk of cancer was 19.6% for one nodule and 36.4% for four or more.

The natural history of these nodules seems to be slow. Thus, two attitudes emerge from the literature: clinical monitoring followed by ultrasound in case of doubtful palpation, or follow-up by regular ultrasound (every 2–5 years) with an annual clinical examination, starting five to eight years after radiation therapy. If nodule(s) have suspicious imaging characteristics (hypoechogenicity, microcalcifications, irregular contours, or mixed types of vascularization (peripheral and central or penetrating radial), cytology is required regardless of the size of the suspect nodule [30, 31]. Also, in the case of malignant cytology in a nodule equal to or greater than 1 cm, a total thyroidectomy is recommended [32]. For isolated nodules less than 1 cm in diameter, there is no demonstration that immediate surgery may be beneficial in terms of cure rate as compared to delayed surgery performed at the time of progression. Therefore, the interest of routine screening for thyroid nodules remains debated. In addition, for patients whose analysis is in favour of a benign nodule but who have a multinodular thyroid, or for patients whose nodules are too small for cytology, regular monitoring is recommended.

In France, the national breast screening programme starts at the age of 50 and is based on mammography every two years. With this in mind, we conducted two studies:

The first was to analyze the characteristics of the secondary breast cancer (SBC) developed in women under the age of 50 (article in press, presented in part at SIOP and ESLCC congress). This was a multicentre retrospective study on 102 women in nine French centres, treated between 1950 and 1995. The median delay from irradiation to SBC was 20 years, and for five women, SBC occurred in the first decade after the first cancer (respective delay: 3, 5, 7, 7, 8 years after); the dose of irradiation was ≥20 Gy in 76% of the cases. The diagnosis of SBC was made at a symptomatic stage in 88.3% of cases (pain, lump, discharge, or skin retraction). Only 23 women had a follow-up focusing on the breasts before SBC diagnosis, either exclusively clinical (4%), or by annual or biennial mammography (13%), or by biennial echography and mammography for a patient at high risk (50 Gy at the level of mediastinum and three relatives with breast cancer). None was followed by MRI.

The second was a survey of current practice on long-term follow-up care (article in press, presented at the SFCE meeting). This survey was sent to every member of the SFCE (French Society for Childhood Cancer) by mail in 2016. Fifty three doctors from 31 centers answered, 18 doctors were used to prescribe breast screening if needed, and 28 were used to prescribe thyroid screening if needed. This survey confirmed that individual thyroid screening was more widespread, probably due to the facility of the screening procedure (non-ionizing exam) (Additional file 1).

France is lacking a specialized screening programme for irradiated childhood survivors, even if physicians are aware of the increased risk and national recommendations have been issued. Long-term follow-up for adults is challenging and requires time, money and organization. In addition, the possible lack of availability of MRI machines has been pointed out.

A study is therefore required to assess, and if possible overcome, all the possible barriers. This is then the main purpose of the DeNaCaPST study, which is supported by the SFCE.

## Methods/design

### Study design and endpoints

DeNaCaPST is a non-interventional study whose aim is to organize a national screening for thyroid cancer and breast cancer in survivors of CAYA cancers. It will study the compliance with international recommendations, with the aim, regarding a breast screening programme, of offering for every woman living in France, at equal risk, an equal screening. Indeed, BRCA-mutated patients are well followed up and they have the same risk as the patients for whom this study is designed.

The primary endpoint is to assess the percentage of patients who will participate in the programme. The secondary endpoints are observance, the number of screened cancers, the number of false positive cases, the number of false negative cases and the usual items considered in screening programmes. Psychological impact, adhesion of the patients in the long term and economic impact will also be studied. A specific survey will also be sent to all investigators to better understand their organization (delay in performing examinations, barriers to screening, etc.).

The primary endpoint is the percentage of persons who participates in the screening programme among the persons at risk with a purpose of rate ≥ 70%. The secondary criteria will take nto account general criteria used in screening cancer programme to assess:

¤ feasibility and efficiency: participation, compliance, number of cases detected, false positives, false negatives, adherence in time to the program

¤ side effects: false positives, psychological impact

¤ results: incidence, characteristics of cancers

¤ costs

### Organization

DeNaCaPST trial is coordinated by the INSERM 1018 unit in cooperation with the LEA (French Childhood Cancer Survivor Study for Leukaemia) study’s coordinators, the long term follow up committee and the paediatric radiation committee of the SFCE. The sponsor of the study is the foundation ARC (Association for Research against Cancer).

A total of 35 centres spread across metropolitan France and la Reunion will participate.

### Patient identification and contact

FCCSS (French Childhood Cancer Survivor Study), LEA and central registry will be interrogated to identify eligible patients [33, 34]. It will be possible for patients to be screened in the center nearest to their current residence, even if they were treated for paediatric cancer in a different network. To participate, centers agreed to perform a complete “long-term follow-up consultations” according to good clinical practice and the guidelines of the SFCE (French Society of Children Cancers) [35]. Consultations will cover explanations of the risk and the appropriate screening programme and, after informed consent, completion of a national socio-psychological questionnaire. Patients will be reviewed at routine follow-up clinics or at recommended practices for the screening.

### Patient selection

Table 1 summarizes the inclusion criteria.

**Table 1:** Inclusion Criteria for DeNaCaPST Programme

	Breast cancer screening	Thyroid cancer screening
Population	Adults treated before the age of 20 years (≤ 20) for a cancer or a leukaemia with an overlap ≥5 years without treatment	
Dose of radiotherapy received for the cancer or the leukaemia	≥ 10 Gy on the mammary bud	≥ 3 Gy on the thyroid
Delay from radiotherapy to inclusion	≥ 8 years	≥ 5 years
Sex and age at time of inclusion	Female ≥ 25 years	Female and Male ≥ 18 years

In practice, the breast screening will be mainly concerned with women treated with a thoracic or mediastinal radiation field, or a TBI ≥ 6 Gy in a single session, or ≥8 Gy in a fractionated treatment. Attention will be paid to patients with an axillary, cervical, cranio-spinal or abdominal field if the distance between the mammary bud and the breast is less than 4 cm. In the case of an abdominal field, mainly girls under the age of 4 would be concerned. Those not of concern, a priori, include facial, cerebral field or metabolic radiation. This means that in this case detailed dosimetric data will be needed.

In practice, for the thyroid screening, patients will be mainly concerned if they have received a mediastinal, cervical, cranio-spinal field, or been treated with a radiation field of 20 Gy or more covering the thyroid (even partially), or whose radiation field limit was supposed to be at 1 cm or less from the thyroid, or at 2 cm or less for higher doses. Those not of concern, a priori*,* include patients treated with an axillary, abdominal, members, cerebral, or facial field. A minority of patients who were treated with a cerebral prophylaxis including the first vertebrae may be at risk.

A centralized agreement will be sent to each investigator after having reviewed data of the first cancer treatment.

### Monitoring

Data monitoring will include dose and date of the radiotherapy, chemotherapy regimen, relapse status, other eventual second malignancies and gynecological status. For data monitoring and organization of the screening, online software has been adapted from that used for BRCA-mutated patients. Data monitoring will be done by the investigators directly for some patients, and for others (in particular, patients included in the LEA cohort or in the FCCSS cohort), data will be already supplied.

### Ethics, informed consent

The final protocol has been approved by the CNIL (French control authority for the protection of personal data), the CCTIRS (Advisory Committee on the Treatment of Research Information in the Field of Health) and ethics committee of the Inserm. Informed consent will be obtained from each patient in oral and written form before inclusion.

### Screening plan

For breast screening, an annual clinical examination is recommended (optional every six months) including the axillary region. Self-palpation is not recommended as the results are not better and it increases stress. The imaging surveillance is based on annual MRI with experienced radiologists (those who propose screening for BRCA-mutated women) with the aim of having a short delay between the radiological examination and the biopsy if needed. Full-field digital mammography is not systematically realized before the age of 30 years (but is recommended for the first screening examination) [27]. To date, after the age of 30 years, one oblique incidence is recommended, but this can change in parallel with the program for women with a constitutional BRCA mutation. Ultrasound is optional and will be done by the radiologist if needed. If several examinations are planned, MRI should be realized first, if possible on the same day and by the same radiological department.

The following examinations depend on the results. The BI-RAD (Breast Imaging Reporting and Data System) classification, established by the American College of Radiology, will be used. If it is normal (BI-RAD 1 and 2), the next follow-up will be one year later. If the results are Bi-RAD 4 or 5, a biopsy will be done. If it is in between (BI-RAD) the following exam will be done 4–6 months later.

For thyroid screening, a clinical exam is recommended every two years (optional every year) including the cervical nodes region. The imaging surveillance is based on ultrasounds realized every two years with experienced radiologists.

The following examinations depend on the results. The TI-RAD (Thyroid Imaging-Reporting and Database System) classification will be used. A FNA (Fine-Needle Aspiration) should be realized in cases of TI-RAD 4A, 4B and 5 over 1 cm and in cases of TI-RAD 3 over 2 cm. If a new nodule is detected or if one changes (but not in case of TI-RAD 4A or 5, where a FNA is needed) the next examination should be one year later.

### Statistics

#### Statistical methods

Descriptive statistics: a descriptive analysis will be carried out integrating the common elements allowing to describe qualitative or quantitative variables (absolute number, percentage, mean, standard deviation, minimum and maximum values, extended ...).

Comparative Statistics: To compare the histological characteristics of the cancers diagnosed with DeNaCaPST with those of the LEA and FCCSS cohorts not included, statistical tests (chi2 or exact Fisher test for low numbers) will be used.

Analytical Statistics: Search for possible causal relationships between treatment received in childhood and time of diagnosis of a second cancer, or between treatment and histological characteristics - Cohort study on risk factors for secondary cancers. Cox proportional hazards models, dose-response modeling using standard epidemiological radio models (linear, linear quadratic, and linear quadratic exponential models) are potentially used. On the available data an estimate of Excess of Relative Risk per unit dose (ERR/Gy) will be calculated.

The primary endpoint concerns the participation rate. A total of about 2000 subjects will be screened for breast and/or thyroid cancer. This workforce will reveal the participation rate with an accuracy of around 2%. If the observed participation rate is 70%, the confidence interval around this percentage will go from 68% to 72%. Such precision is not necessary, but the enumeration included will allow to study the predictive factors of participation rate, which are not known, and to have a reasonable precision, even in the analysis of subgroups.

## Discussion

International guidelines have been put in place because of the evidence for increased lifetime risk of breast and thyroid cancer. DeNaCaPST is based on these international recommendations but it is important to recognize that they are based on expert consensus opinion and are supported by neither nonrandomized observational studies nor prospective randomized trials in this specific population. In the general population, some studies have been designed as blind and randomized studies, but we have to point out that in the field of breast cancer screening data are sometimes distorted or inappropriately used, and that screening is also a theme of passionate debate [36]. The main goal of cancer screening is to reduce the specific mortality from cancer. But expected mortality depends strongly on the mortality rate, which evolves with time. In breast cancer, mortality rate is reducing because of the growing effectiveness of treatments [37]. Thus the endpoint of screening studies should not focus only on reducing specific mortality [38], and the overall effectiveness of a screening program (including the risk of over-diagnosis) can be definitively assessed only through randomized controlled trials. Nevertheless those trials are ethically questionable.

Over-diagnosis is a phenomenon inherent in any screening program. This means that clinicians must be aware of the problem and such programs must be regularly reviewed, given such therapeutic advances and new studies produced, to re-examine the real impact of any program on mortality and the extent of its deleterious effects. Indeed, as it is not possible to predict the evolution of a cancerous lesion at the time it is detected, it is proposed to treat all detected cancers, but this may result in overtreatment for cancers that would have not evolved through time.

Evaluation of over-diagnosis is possible only through randomized studies comparing screened and non-screened populations. Otherwise it must employ assumptions modeled from the rate of non- progressive cancers. The results published for this kind of analysis vary greatly depending on the type of cancer concerned (eg. in breast “infiltrating only” or “infiltrating + in situ”), the methodology, assumptions, indicators and parameters chosen.

For micro-papillary thyroid cancer of less than 1 cm in diameter when they are isolated (without lymph node metastases and without extra-capsular extension), there is no data demonstrating a benefit of immediate surgery as compared to delayed surgery performed at progression. Therefore a programme of active surveillance may be offered to these patients, that may be combined to translational research to discover biological markers that are predictive of future progression.

As survival has greatly improved in childhood cancers, detection of therapy-related malignancies has become a priority even if new radiation techniques will lead to better protection for organs at risk. DeNaCaPST is a multicentre study whose aim is to prove that a screening for thyroid and breast cancers is possible at a national level for at-risk survivors. We hope that it will result in a similar follow-up for those at equal risk across the country. It will also provide additional data on secondary thyroid and breast cancers, and reinforce multidisciplinary cooperation.

## Additional files

Additional file 1: Document 2. Survey about LTFU care and specific questions about second cancers. (DOCX 13 kb)

Additional file 2: Document 1. List of involved centers or hospitals. (DOCX 12 kb)

## Abbreviations

ACS: American Cancer Society
BI-RAD: Breast Imaging-Reporting and Database System
CAYA: Childhood adolescente young adult
CCTIRS: Advisory Committee on the Treatment of Research Information in the Field of Health
CNIL: French control authority for the protection of personal data
COG: Children’s Oncology Group
ERR: Excess of Relative Risk
FCCSS: French Childhood Cancer Survivor Study
FNA: Fine-Needle Aspiration
Gy: Gray
HAS: French High Authority of Health
LEA: French Childhood Cancer Survivor Study for Leukaemia
MRI: Magnetic resonance imaging
NCI: National Cancer Institute
SBC: Second breast cancer
SFCE: French Society of Childhood Cancers
TI-RAD: Thyroid Imaging-Reporting and Database System
UK CCSG: Children’s Cancer and Leukaemia Group
