# Supplementary material for: A French national breast and thyroid cancer screening programme for survivors of childhood, adolescent and young adult (CAYA) cancers - DeNaCaPST programme
Source: BMC Cancer. 2017 May 12;17:326. doi: 10.1186/s12885-017-3318-1 (PMC5427546; doi:10.1186/s12885-017-3318-1)
Supplement: Supplementary file 1 — Survey about LTFU care and specific questions about second cancers. (DOCX 13 kb) [file 12885_2017_3318_MOESM2_ESM.docx]

Participating centers are French centers :

| ICO Nantes |
| --- |
| CHU Nantes |
| CHU Nancy |
| ICL Nancy |
| Institut Gustave Roussy Villejuif |
| Institut curie Paris |
| CHU Saint Etienne |
| Centre Léon Bérard Lyon |
| Centre Paul Strauss Strasbourg |
| Centre Oscar Lambret Lille |
| CHU Lille |
| CHU Rennes |
| Centre Eugène Marquis Rennes |
| Centre Eugène Marquis |
| CHU - Centre la Timone Marseille |
| CHU- Hôpital d’enfants Marseille |
| CHU Bordeaux |
| IUCT-oncopole Toulouse |
| CHU-Toulouse |
| Institut Val d’Aurelle Montpellier |
| CHU Arnaud de Villeneuve Montpellier |
| Centre Antoine Lacassagne Nice |
| CHU Nice |
| Centre Baclesse Caen |
| CHU Caen |
| CHU Rouen |
| CLCC Becquerel Rouen |
| Institut Jean Godinot Reims |
| CHU Reims |
| CHU Limoges |
| CHU Dijon |
| Centre Georges François Leclerc Dijon |
| CHU Angers |
| CHU Grenoble |
| CHU Estaing Clermont Ferrand |
| CHU St Denis de la Réunion |
