# Supplementary material for: A French national breast and thyroid cancer screening programme for survivors of childhood, adolescent and young adult (CAYA) cancers - DeNaCaPST programme
Source: BMC Cancer. 2017 May 12;17:326. doi: 10.1186/s12885-017-3318-1 (PMC5427546; doi:10.1186/s12885-017-3318-1)
Supplement: Supplementary file 2 — List of involved centers or hospitals. (DOCX 12 kb) [file 12885_2017_3318_MOESM1_ESM.docx]

# Long Term Follow-Up (LTFU) Survey

The survey was designed to give answers to these following points :

Identification (1 question)

Knowledge about LTFU and LTFU committee (3 questions)

Existence of LTFU care in the city where you work (1 question)

Definition of LTFU care and LTFU objectives (4 questions)

Habits of follow up after cancer treatment (2 questions)

Organization of follow-up of patients who became adults (15 questions)

About second cancers and screenings (4 questions, see below)

Difficulties about LTFU care (1 question)

Optimal organization of LTFU care (1 open question)

Questions about second cancers:

- « do you prescribe a specific screening ?
- If yes, for which cancer ? breast ? thyroid ? lung . colo-rectum ? other ?
- If yes, could you detail the exams and frequency ?
- Do you have some problems concerning the organization of the screening ?”
